# The impact of recipient age on the effects of umbilical cord mesenchymal stem cells on HBV-related acute-on-chronic liver failure and liver cirrhosis

**DOI:** 10.1186/s13287-021-02544-x

**Published:** 2021-08-20

**Authors:** Ka Zhang, Haixia Sun, Huijuan Cao, Yifan Jia, Xin Shu, Hong Cao, Yufeng Zhang, Xiaoan Yang

**Affiliations:** Department of Infectious Diseases, The Third Affiliated Hospital of Sun Yat-Sen University, Guangzhou, People’s Republic of China

**Keywords:** Umbilical cord mesenchymal stem cell transplantation, Liver failure, Liver cirrhosis, Hepatitis B virus, Age factors

## Abstract

**Background:**

The results of a previous study verified that umbilical cord mesenchymal stem cells (UCMSCs) have good therapeutic effects for the treatment of HBV-related acute-on-chronic liver failure (ACLF) and liver cirrhosis (LC). Nevertheless, it is still unknown whether the effects of UCMSCs are affected by recipient age.

**Methods:**

Patients treated with UCMSCs who met the criteria of HBV-related ACLF and liver cirrhosis were identified in this retrospective observational study. Patients were divided into subgroups according to the World Health Organization (WHO) age criteria (< 45 vs. ≥ 45 years). Group A included young ACLF patients (< 45 y), and group B included older ACLF patients (≥ 45 y). Young LC patients (< 45 y) were assigned to group C, and group D included older LC patients (≥ 45 y). Patients’ clinical characteristics, demographics, biochemical factors, and model for end-stage liver disease (MELD) scores were compared for 24 weeks.

**Results:**

Sixty-four ACLF patients and 59 LC patients were enrolled in this study. Compared with patients in groups B and C, patients in group A did not show significant superiority in terms of the levels of ALT, AST, TBIL, AFP, and PTA and MELD scores. However, the median decrease and cumulative decrease in the TBIL and ALT levels of patients in group C were larger than those of patients in group D after four weeks of UCMSC transfusions. For older patients (≥ 45 y), the cumulative decrease and the median decrease in the TBIL of ACLF patients were significantly greater than those of LC patients after UCMSC treatment. However, the median decrease in ALT levels of ACLF patients was significantly greater than that of LC patients during UCMSC treatment, and the cumulative decrease in ALT levels of ACLF patients was significantly greater than that of LC patients at all time points.

**Conclusion:**

The therapeutic effects of UCMSCs for HBV-related acute-on-chronic liver failure and liver cirrhosis varied partly by patient age. Assessing patient age is necessary prior to UCMSC clinical use.

**Supplementary Information:**

The online version contains supplementary material available at 10.1186/s13287-021-02544-x.

## Introduction

Acute-on-chronic liver failure (ACLF) remains a global health concern. The latest research confirms that the global prevalence and mortality rate of ACLF are high, and the global prevalence of ACLF among patients admitted with decompensated cirrhosis was 35%; its prevalence is highest in South Asia (65%) [1]. Hepatitis B virus (HBV) infection is the most common cause of ACLF and cirrhosis. Thus, HBV-related end-stage liver disease mainly includes liver failure and decompensated cirrhosis. Because of its rapid progression and poor prognosis, the only curative therapy for end-stage liver disease is orthotopic liver transplantation (OLT) [2]. A shortage of donor livers, risks of transplantation and long-term use of immunosuppressants after transplantation limit the application of liver transplantation [3]. Therefore, it is particularly urgent to search for new treatments for patients with HBV-related end-stage liver disease.

In theory, cell therapy has great potential for patients with end-stage liver disease. Cell therapy, including mesenchymal stem cell (MSC) and macrophage cell therapy, can be used to replenish liver cells or to remodel and repair the damaged liver [4]. Many basic and clinical studies have provided evidence that MSCs are safe and effective for the treatment of liver failure and cirrhosis [5–7]. The results of previous studies from our department showed that allogeneic bone marrow-derived MSCs are effective and safe for HBV-related ACLF patients [8, 9]. In our last study, we observed that umbilical cord mesenchymal stem cells (UCMSCs) also showed good therapeutic effects for HBV-related ACLF and liver cirrhosis, and this therapeutic effect could be enhanced by prolonging the UCMSC treatment course, especially for patients with cirrhosis [10]. However, a series of factors influencing the therapeutic effects of MSCs during the treatment of liver failure and cirrhosis have also been proposed by other scholars, such as the type of MSCs, the method of infusion, the dosage of infusion, and the time of infusion [11]. Nevertheless, almost all studies have focused on only factors related to MSCs. Less attention has been given to the recipient factors influencing the therapeutic effects of MSCs.

The influence of MSC recipient age cannot be avoided in clinical research on UCMSC treatment for HBV-related ACLF and liver cirrhosis. There are limited data available on the effect of recipient age on the therapeutic effects of MSCs. Irma Virant-Klun et al. [12] reported that age in females can significantly influence the pluripotency of MSCs, expression of MSC-related genes, and MSC differentiation potential. Animal experiments have demonstrated that neural stem cell (NSC) survival is dependent on the sex and age of the recipient [13]. Adult stem cells play a vital role in preventing the aging of organs and tissues and can delay aging [14]. Adult stem cells also undergo some detrimental changes during aging, such as alterations in the microenvironment, a decline in regenerative capacity, and loss of function [14].

Here, we explored whether recipient age affects the therapeutic effects of UCMSCs during the treatment of HBV-related ACLF and cirrhosis. Our results indicate that the therapeutic effects of UCMSCs for HBV-related ACLF and cirrhosis varied partly by patient age.

## Methods

### Study population

We collected data from HBV-related ACLF or LC patients who received UCMSCs at the Third Affiliated Hospital of Sun Yat-sen University between February 2014 and December 2015. The study was approved by the Human Ethics Committee of The Third Affiliated Hospital of Sun Yat-sen University, Guangzhou, China. Our study procedures adhered to the tenets of the Declaration of Helsinki, and informed consent was obtained from all patients. LF patients who met the 2009 APASL diagnostic criteria for hepatitis B liver failure and cirrhosis [15] and ACLF patients who met the criteria outlined in the Guidelines for Diagnosis and Treatment of Liver Failure (China, 2018 Edition) [16] were deemed eligible for study enrollment. The brief ACLF diagnostic description is as follows: ACLF can be divided into three types (A, B and C): Type A: ACLF based on chronic noncirrhotic liver disease; Type B: ACLF based on compensated cirrhosis; and Type C: ACLF based on decompensated cirrhosis. We selected ACLF of type A in this study. The exclusion criteria of the patients were as previously described [10].

### Study design

First, patients with HBV-related end-stage liver disease treated with UCMSCs were included in this study. The patients were divided into two groups according to their diagnosis: the ACLF group and liver cirrhosis group (LC). Second, according to the criteria of the WHO in 2012 [17], younger age was defined as less than 45 years (< 45 y), and older age was defined as greater than or equal to 45 years (≥ 45 y). Based on these definitions, the ACLF group was then categorized into group A and group B. Group A (< 45 y) included liver failure patients younger than 45 years, and group B (≥ 45 y) included liver failure patients aged 45 years or older. The LC group was divided into group C, including patients younger than 45 years (< 45 y), and group D (≥ 45 y), including patients aged 45 years or older. Finally, the patients were followed up for 24 weeks. Data from all patients were collected at baseline and at 1, 4, 12 and 24 weeks after therapy. Additionally, all patients in this study received standard clinical treatments (including coagulation correction, albumin supplementation, antiviral treatment, S-adenosylmethionine infusion, and necessary anti-infection treatment) before stem cell infusion. All patients were provided with relevant information on stem cell treatment and signed the informed consent form before receiving stem cell treatment. Statistically, the levels of glutamic-oxaloacetic transaminase (AST), alanine aminotransferase (ALT), prothrombin activity (PTA), alpha fetoprotein (AFP), and total bilirubin (TBIL) and the model for end-stage liver disease (MELD) scores were comprehensively analyzed to evaluate the effects of recipient age on the treatment effect of UCMSCs.

### UCMSC preparation and transfusion

The processing of the umbilical cords and preparation of UCMSCs were performed at the GMP Stem Cell Laboratory Facility of the Biotherapy Center of The Third Affiliated Hospital of Sun Yat-sen University, Guangzhou, China. The specific details of the isolation, culture and characterization of UCMSCs were described in a previous report [18]. Quality control was performed as described in our previous study [10]. Patients received UCMSC transfusions at 1, 2, 3 and 4 weeks after recruitment. As detailed in our previous research, approximately 1.0 × 10^6^ UCMSCs per kilogram of body weight suspended in 100 mL of normal saline solution were infused intravenously through a forearm vein at each treatment time [10].

### Statistical analysis

Continuous variables are expressed as the mean ± standard deviation (SD) or median (interquartile) depending on the results of normality testing and were analyzed using the t-test or Wilcoxon test, as appropriate. Normality analysis was performed by the Shapiro–Wilk test. Normally distributed variables were analyzed with ANOVA, while nonnormally distributed variables were analyzed with nonparametric tests. Sex was expressed as the number of patients (percentage) and analyzed with the χ2 test or Fisher’s exact test, as appropriate. SPSS software (version 22.0; SPSS, Inc., Chicago, IL) was used to perform the statistical analyses. All analyses were performed as two-sided tests with a 0.05 level of significance.

## Results

### Patient characteristics

According to the diagnostic criteria, 64 ACLF patients and 59 liver cirrhosis patients were enrolled and eligible for efficacy analysis in this study. According to the age criteria of the WHO, the ACLF group was again divided into two subgroups: group A (age < 45 y, *N* = 37) and group B (age ≥ 45 y, *N* = 27). The liver cirrhosis group was also divided into two subgroups: group C (age < 45 y, *N* = 27) and group D (age ≥ 45 y, *N* = 32). The baseline characteristics of these patients are shown in Tables 1 and 2. The variables of group A and group B were generally similar, and no significant differences were observed between the two groups (Table 1). However, the differences between group C and group D, group A and group C, and group B and group D were statistically significant (Tables 1 and 2).

**Table 1:** Comparison of patient demographics and baseline characteristics between group A and group B and between group C and group D

	Liver failure			Liver cirrhosis		
	Group A (Age < 45)	Group B (Age ≥ 45)	P value	Group C (Age < 45)	Group D (Age ≥ 45)	P value
Sex (M/F)	33/4	23/4	0.712	25/2	24/8	0.092
Age (years)	35 (31.5 to 40)	49 (46 to 61)	0.000	36 (33 to 40)	53.5 (50.2 to 65)	0.000
ALT (U/L)	110 (51 to 233.5)	71 (42 to 395)	0.430	66 (33 to 225)	38.5 (18.5to 52.8)	0.001
AST(U/L)	104 (73.5 to 155.5)	96 (59 to 161)	0.321	81 (66 to 122)	55.5 (32.5 to 82.5)	0.002
TBIL (mmol/L)	419.3 ± 167.0	379.9 ± 182.5	0.369	334 (158 to 487)	120.5 (53.8 to 444.5)	0.024
PTA	34 (27 to 42)	35 (28 to 43)	0.600	42 (34 to 48)	43 (34 to 59)	0.645
MELD	27 (25 to 30)	27 (25 to 30)	0.881	25 (21 to 26)	22 (17 to 26)	0.155
AFP	105.7 (36.5 to 219.7)	70.5 (7.5 to 200.6)	0.229	85.0 (8.1 to 290.0)	7.8 (1.9 to 38.0)	0.002

*ALT* alanine aminotransferase, *AST* glutamic-oxaloacetic transaminase, *TBIL* total bilirubin, *PTA* prothrombin time activity, *MELD* model for end-stage liver disease, AFP alpha fetoprotein

**Table 2:** Comparison of patient demographics and baseline characteristics between group A and group C and between group B and group D

	Age ≤ 45			Age > 45		
	Group A Liver failure	Group C Liver cirrhosis	P value	Group B Liver failure	Group D Liver cirrhosis	P value
Sex (M/F)	33/4	25/2	1.000	23/4	24/8	0.092
Age (years)	35.11 ± 4.40	35.85 ± 4.80	0.523	49 (46 to 61)	53.5 (50.3 to 65)	0.053
ALT (U/L)	110 (51 to 233.5)	66 (33 to 225)	0.157	71 (42 to 395)	38.5 (18.5 to 52.8)	0.000
AST (U/L)	104 (73.5 to 155.5)	81 (66 to 122)	0.062	96 (59 to 161)	55.5 (32.5 to 82.5)	0.002
TBIL (mmol/L)	419.3 ± 167.0	317.96 ± 179.28	0.023	407 (248 to 503)	120.5 (53.8 to 444.5)	0.002
PTA	34 (27 to 42)	42 (34 to 48)	0.013	35 (28 to 43)	43 (34 to 59)	0.022
MELD	27 (25 to 30)	25 (21 to 26)	0.002	27 (25 to 30)	22 (17 to 26)	0.000
AFP	105.7 (36.5 to 219.7)	85.0 (8.1 to 290.0)	0.714	70.5 (7.5 to 200.6)	7.8 (1.9 to 37.9)	0.011

*ALT* alanine aminotransferase, *AST* glutamic-oxaloacetic transaminase, *TBIL* total bilirubin, *PTA* prothrombin time activity, *MELD* model for end-stage liver disease, *AFP* alpha fetoprotein

### Comparative analysis of the UCMSC therapeutic effect for ACLF patients in group A and group B

**Fig. 1:**
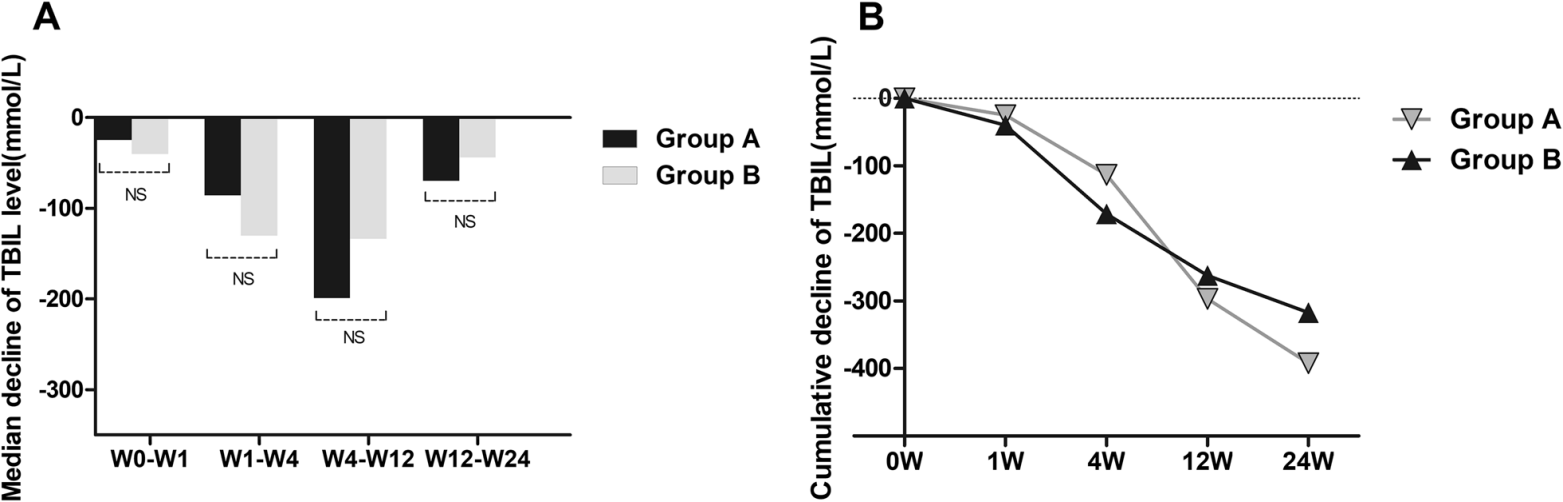
Comparison of the therapeutic effect of UCMSCs between younger liver failure patients and non-liver failure patients among different observation weeks. Group A: liver failure patients (age < 45 years); Group B: liver failure patients (age ≥ 45 years); *TBIL* total bilirubin. ***p < 0.01, **p < 0.05

The therapeutic effect of UCMSCs for ACLF patients varied based on the age of patients in the ACLF group. To understand whether the age of patients affects the efficacy of UCMSCs, we compared the outcome of group A (age < 45 y) with that of group B (age ≥ 45 y) in patients with acute-on-chronic liver failure. The median decrease in serum TBIL showed no difference between group A (age < 45 y) and group B (age ≥ 45 y) at any of the different time points (W0–W1, W1–W4, W4–W12, and W12–W24) (Fig. 1a). Of interest, the median decrease in TBIL gradually increased in group A after four weeks of treatment, but no statistically significant differences were maintained (Fig. 1a). Likewise, when the cumulative decrease in TBIL was compared between the two groups at different time points, no significant difference was found at any observation week (W1, W4, W12, and W24) (Fig. 1b). The median or cumulative decrease in the ALT, AST, TBIL, AFP values and MELD scores showed no difference between group A (age < 45 y) and group B (age ≥ 45 y) at any of the different time points (Additional file 1: Table S1 and Table S2).

### Comparative analysis of the therapeutic effect of UCMSCs on liver cirrhosis patients in groups C and D

**Fig. 2:**
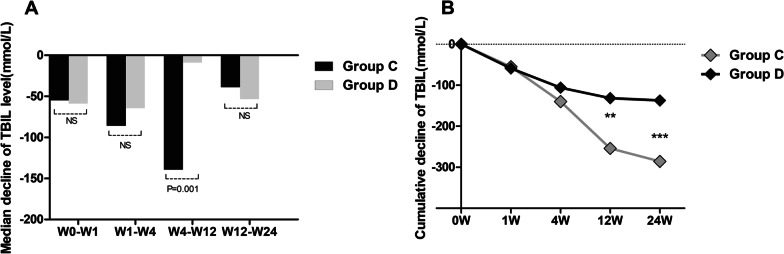
Comparison of the therapeutic effect of UCMSCs between younger liver cirrhosis patients and older liver cirrhosis patients at different observation weeks. Group C: liver cirrhosis patients (age < 45 years); Group D: liver cirrhosis patients (age ≥ 45 years); *TBIL* total bilirubin. ***p < 0.01, **p < 0.05

To understand whether the age of patients affects the efficacy of UCMSCs in liver cirrhosis patients, we compared the outcome of group C (age < 45 y) with that of group D (age ≥ 45 y), and the efficacy varied based on patient age. The median decrease in serum TBIL showed no difference between group C (age < 45 y) and group D (age ≥ 45 y) at W0–W1, W1–W4, and W12–W24 (Fig. 2a). However, patients aged less than 45 years (age < 45 y, group C) had larger decreases in TBIL levels than patients aged 45 years or older (age ≥ 45 y, group D) after four UCMSC transfusions (at W4–W12) (Fig. 2a). When the cumulative decrease in TBIL was compared between the two groups at different time points, no significant difference was found at W1 and W4 (Fig. 2b). Surprisingly, the cumulative decrease in TBIL of group C (age < 45 y) gradually increased from week 4 and was larger than that of group D (age ≥ 45 y) (p < 0.05) (Fig. 2b). The median and cumulative decreases in the ALT levels of group C (age < 45 y) were larger than those of group D (age ≥ 45 y) at W1–W4 and W4 (*p* < 0.05) (Tables 2 and 3). However, no apparent difference was found at the other three time points (Tables 2 and 3). Similarly, the median decrease in the MELD score of group C was also larger than that of group D at W4–W12, and the cumulative decrease was larger than that of group D at W24 (*p* < 0.05) (Tables 3 and 4). Regarding AFP levels, a difference was only found in the first week (Tables 3 and 4). Finally, no statistically significant changes in the levels of AST and PTA were found between the two groups at any of the four time points.

**Table 3:** Comparative analysis of the therapeutic effect of UCMSCs on the median decrease in various parameters

Outcome	Group	W0–W1		W1–W4		W4–W12		W12–W24	
		Value	P value	Value	P value	Value	P value	Value	P value
AST	Group C	13 (− 10.5 to 40)	NS^CD^	8 (− 12.3 to 24.5)	NS^CD^	10 (2.5 to 26)	NS^CD^	9.5 (− 0.3 to 17.5)	NS^CD^
	Group D	5 (0 to 27.5)		− 1.5 (− 12.5 to 13.8)		7.5 (− 5.8 to 37.5)		− 3 (− 24 to 7)	
	Group B	22 (4 to 104.3)	NS^BD^	4 (− 14 to 25)	NS^BD^	17.5 (− 10.3 to 42)	NS^BD^	2 (− 5 to 17.5)	NS^BD^
ALT	Group C	20 (− 3.5 to 153.5)	NS^CD^	14.5 (2 to 21.5)	0.006^CD^	1 (− 7 to 6.5)	NS^CD^	− 0.5 (− 6.5 to 6.8)	NS^CD^
	Group D	7 (2 to 23.25)		− 2 (− 5.8 to 4)		1.5 (− 3.8 to 6.25)		2 (− 15 to 7)	
	Group B	32.5 (9.8 to 335.5)	0.005^BD^	15 (− 2.5 to 27.5)	0.007^BD^	5.5 (− 9 to 18.5)	NS^BD^	0 (− 4 to 5.5)	NS^BD^
PTA	Group C	0.5 (− 6.5 to 6.8)	NS^CD^	2 (− 1 to 8.5)	NS^CD^	7 (3 to 17)	NS^CD^	6 (5.5 to 19)	NS^CD^
	Group D	0.5 (− 5.3 to 4)		4 (− 2 to 12)		4.5 (− 3 to 10.5)		− 1 (− 2.5 to 0.5)	
	Group B	− 1.5 (− 5.25 to 2)	NS^BD^	1 (0 to 6)	NS^BD^	7 (0 to 14)	NS^BD^	3.5 (− 1 to 13)	NS^BD^
MELD	Group C	0.5 (− 2 to 4)	NS^CD^	1 (− 0.5 to 3)	NS^CD^	6 (1 to 10)	0.020^CD^	3 (2.5 to 7.5)	NS^CD^
	Group D	1 (− 1 to 3)		2 (1 to 4)		0.5 (− 1.3 to 4.3)		− 1 (− 1 to 6.3)	
	Group B	0 (− 1 to 2)	NS^BD^	3 (0 to 4)	NS^BD^	5.5 (1 to 8.8)	0.030^BD^	− 1 (− 1.1 to 6)	NS^BD^
AFP	Group C	32 (3 to 255)	0.021^CD^	− 2 (− 6 to 30)	NS^CD^	− 2 (− 36.8 to 12.5)	NS^CD^	40.5 (15 to 66)	NS^CD^
	Group D	4.5 (1 to 27.8)		− 1 (− 22 to 0.5)		0.5 (− 3.8 to 6.3)		0 (− 7.8 to 2.5)	
	Group B	9 (1 to 85)	NS^BD^	− 10 (− 76 to 81)	NS^BD^	3 (− 8 to 4)	NS^BD^	0 (− 2 to 0)	NS^BD^

*ALT* alanine aminotransferase, *AST* glutamic-oxaloacetic transaminase, *TBIL* total bilirubin, *PTA* prothrombin time activity, *MELD* model for end-stage liver disease, *AFP* alpha fetoprotein. BD group B versus group D, CD group C versus group D. *p* ≤ 0.05 was considered to be statistically significant

**Table 4:** Comparative analysis of the therapeutic effect of UCMSCs on the cumulative decrease in various parameters

Outcome	Group	W1–W0		W4–W0		W12–W0		W24–W0	
		Value	P value	Value	P value	Value	P value	Value	P value
AST	Group C	13 (− 10.5 to 40)	NS^CD^	16 (− 20 to 57)	NS^CD^	27 (− 1 to 77)	NS^CD^	33.5 (9.5 to 85.2)	NS^CD^
	Group D	5 (0 to 27.5)		− 1 (− 6 to 49.5)		5 (− 10.5 to 68)		1 (− 7 to 14.5)	
	Group B	22 (4 to 104.3)	NS^BD^	4 (− 14 to 25)	NS^BD^	17.5 (− 10.2 to 42)	NS^BD^	2 (− 5 to 17.5)	0.004^BD^
ALT	Group C	20 (− 3.5 to 153.5)	NS^CD^	50 (7 to 172)	0.016^CD^	35 (0 to 178)	NS^CD^	54.5 (− 8 to 193)	NS^CD^
	Group D	7 (2 to 23.3)		6.5 (− 1.3 to 36)		9 (− 6.5 to 43.5)		2 (− 10 to 19.5)	
	Group B	32.5 (9.8 to335.5)	0.005^BD^	45 (11 to 360.5)	0.004^BD^	46 (11.5 to 358.8)	0.004^BD^	49 (5 to 402)	0.002^BD^
PTA	Group C	0.5 (− 6.5 to 6.8)	NS^CD^	0.5 (− 8 to 9)	NS^CD^	10.5 (− 0.5 to 27.8)	NS^CD^	0.5 (− 28 to 29.8)	NS^CD^
	Group D	0.5 (− 5.3 to 4)		5.5 (− 4.5 to 14)		4 (− 3.8 to 10.5)		1 (− 3 to 14.5)	
	Group B	− 1.5 (− 5.3 to 2)	NS^BD^	2 (− 6 to 7)	NS^BD^	5 (− 3 to 15)	NS^BD^	1.5 (− 3 to 15.8)	NS^BD^
MELD	Group C	0.5 (− 2 to 4)	NS^CD^	− 0.5 (− 2 to 4.3)	NS^CD^	6.5 (2 to 1.5)	NS^CD^	11 (8 to 12)	0.036^CD^
	Group D	1 (− 1 to 3)		3 (0.8 to 6)		1.5 (− 0.8 to 8.5)		3 (0 to 5.5)	
	Group B	0 (− 1 to 2)	NS^BD^	4 (− 1.5 to 6)	NS^BD^	4 (0.3 to 9.3)	NS^BD^	3 (− 1 to 13)	NS^BD^
AFP	Group C	32 (3 to 255)	0.021^CD^	6 (− 2 to 41)	NS^CD^	1 (− 9 to 59)	NS^CD^	59 (1.5 to 339)	NS^CD^
	Group D	4.5 (1 to 27.8)		0 (− 1 to 3.5)		0 (0 to 22)		0.5 (− 0.8 to 21.8)	
	Group B	9 (1 to 85)	NS^BD^	87 (− 8 to 185)	NS^BD^	9 (− 2.5 to 165)	NS^BD^	20 (0 to 188)	NS^BD^

*ALT* alanine aminotransferase, *AST* glutamic-oxaloacetic transaminase, *TBIL* total bilirubin, *PTA* prothrombin time activity, *MELD* model for end-stage liver disease, *AFP* alpha fetoprotein. BD group B versus group D, CD group C versus group D. *p* ≤ 0.05 was considered to be statistically significant

### Comparative analysis of the therapeutic effect of UCMSCs between younger ACLF patients (age < 45 y) (group A) and younger liver cirrhosis patients (age < 45 y) (group C)

**Fig. 3:**
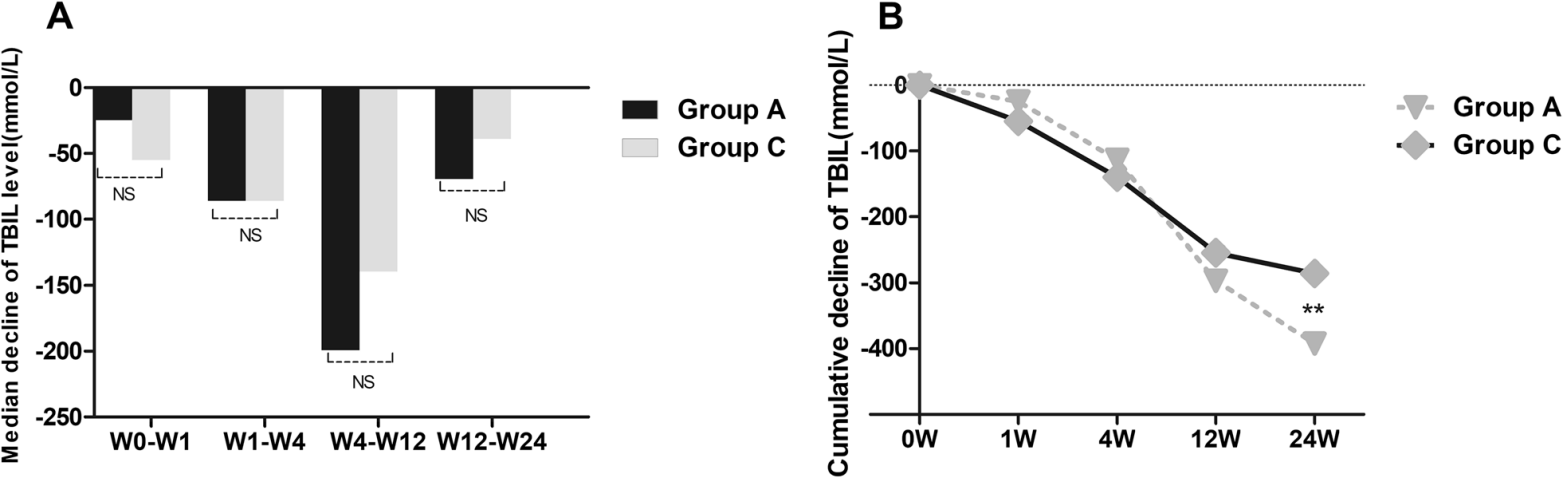
Comparison of the median and cumulative decreases in TBIL levels between group A and group C at different time points. **a** Median decrease in TBIL levels between group A and group C. **b** Cumulative decreases in TBIL levels between group A and group C. Group A: liver failure patients (age < 45 years); Group B: liver cirrhosis patients (age < 45 years); *TBIL* total bilirubin. ***p < 0.01, **p < 0.05

To investigate the therapeutic effect of UCMSCs on younger patients with ACLF failure or liver cirrhosis, we compared the outcome of group A with that of group C. The median decrease in serum TBIL showed no difference between group A and group C at any of the time points (Fig. 3a). When the cumulative decrease in TBIL was compared between the two groups at different time points, no significant difference was found between the two groups at any observation week except week 24 (Fig. 3b). The cumulative decrease in TBIL of patients in group A was higher than that of patients in group C at week 24 (Fig. 3b). The median and cumulative decreases in serum ALT, AST, and PTA values, MELD scores and AFP levels at all post-baseline time points were not significantly different between group A and group C (Additional file 1: Table S3 and Table S4).

### Comparative analysis of the therapeutic effect of UCMSCs between older ACLF patients (age ≥ 45 y) (group B) and older liver cirrhosis patients (age ≥ 45 y) (group D)

**Fig. 4:**
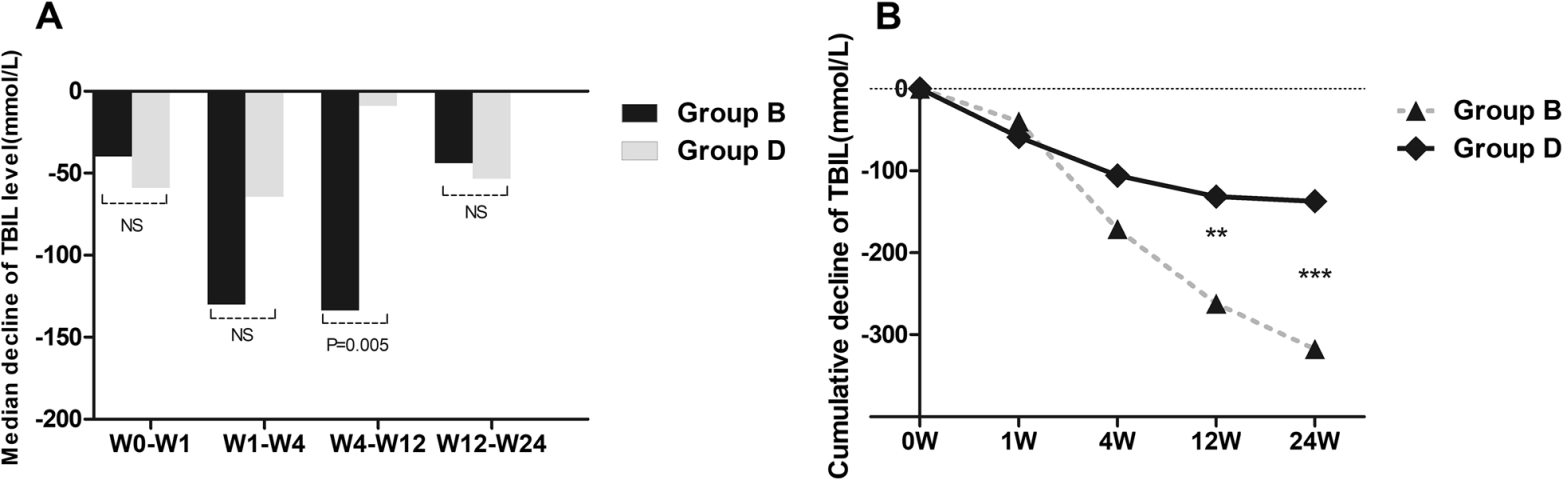
Comparison of the median and cumulative decreases in TBIL levels between group B and group D at different time points. **a** Median decrease in TBIL levels between group B and group D. **b** Cumulative decreases in TBIL levels between group B and group D. Group B: liver failure patients (age ≥ 45 years); Group D: liver cirrhosis patients (age ≥ 45 years); *TBIL* total bilirubin. ***p < 0.01, **p < 0.05

To learn more about the therapeutic effect of UCMSCs in older patients with HBV-related ACLF and liver cirrhosis, the outcomes of patients aged 45 years or older in the ACLF group (group B) were compared with those of older patients with liver cirrhosis (group D). The median decrease in serum TBIL showed no difference between groups B and D at any of the time points except W4–W12 (Fig. 4a). Of interest, the median decrease in TBIL gradually increased in group B after UCMSC treatment, and statistically significant differences were maintained at W4–W12 (Fig. 4a). The cumulative decrease in the TBIL of patients in group B was significantly greater than that of group D at weeks 12 and 24 (Fig. 4b). The AST, PTA, and AFP levels and the MELD scores at all post-baseline time points were not significantly different between groups B and D (Tables 3 and 4). However, the median decrease in the ALT level of patients in group B was significantly greater than that of group D during the first four weeks (W0–W1 and W1–W4) (*p* < 0.05) (Table 3). Likewise, the cumulative decrease in the ALT level of patients in group B was significantly greater than that of group D at all time points (*p* < 0.05) (Table 4).

## Discussion

Because of the discrepancies in liver transplant supply and demand, liver transplantation, which is the gold standard therapy for HBV-related end-stage liver disease, cannot be widely applied in clinical practice. Stem cell transplantation is an alternative option for liver transplantation in HBV-related end-stage liver disease patients [19]. A large-scale meta-analysis of randomized controlled trials (RCTs) evaluating the therapeutic effects and safety of stem cell therapy for chronic liver disease (CLD) revealed that stem cell therapy is a safe and effective therapeutic option for CLD and that patients with ACLF benefit the most in terms of improved short-term survival rates [20]. The results of our previous research showed that UCMSCs also have good therapeutic effects for HBV-related ACLF and liver cirrhosis and that their therapeutic effect could be enhanced by prolonging the UCMSC treatment course [10]. MSCs can promote liver regeneration and repair liver injury by cell migration into liver sites, hepatogenic differentiation, immunoregulation, and paracrine mechanisms [21]. In vivo, MSCs exert immunomodulatory, anti-inflammatory, antifibrotic, antioxidative and antiapoptotic effects on liver cells [22]. However, there are more challenges to be resolved, including determination of the best stem cell source, the optimal route for stem cell transplantation, and the dose and frequency of injected stem cells [23]. Unfortunately, almost all studies have focused only on factors related to MSCs, and less attention has been given to the recipient factors influencing the therapeutic effects of MSCs. After culture and isolation in vitro, deprivation of oxygen and nutrients and a lack of external growth factors are challenges that can influence the efficacy of MSCs [24]. Moreover, after MSCs are injected and migrate into damaged tissues or organs, a harsh environment coupled with death signals due to the inadequate tensegrity structure between the cells and the matrix can influence the efficacy of MSCs [25]. Thus, the obstacle facing MSC‐based transplantation therapy is the limited number of functional stem cells available after transplantation due to the harsh microenvironment, anoikis and inflammation induced by damaged tissues or organs [26]. In short, the microenvironment of MSC recipients influences the efficacy of MSC‐based transplantation therapy. Recipient factors that may influence the therapeutic effects of MSCs need to be taken into consideration.

It has been well established that age has a profound influence on the liver microenvironment. In an earlier study, microarray data showed that inflammation-related gene expression increased with age in the liver [27]. Other researchers [28] investigated immune-related changes in the aged liver and found that the levels of inflammatory cytokines, chemokines, and inflammatory genes were higher in aged animals. The latest results [29] revealed that older age was associated with increased hepatic accumulation of Kupffer and CD11b+ cells, as well as with adaptive immune activation and clinical evolution in chronic hepatitis B associated with age-associated changes in intrahepatic immune subsets. In this study, we mainly focused on whether the effects of UCMSCs on patients with HBV-related acute-on-chronic liver failure and liver cirrhosis were affected by recipient age. In HBV-related ACLF patients, the younger patients did not show significant superiority over the older patients with respect to ALT, AST, TBIL, AFP, and PTA values and MELD scores. However, compared with older liver cirrhosis patients, younger liver cirrhosis patients had distinct advantages. Specifically, a decrease in ALT levels during UCMSC treatment was observed, and the most significant bilirubin decline occurred after UCMSC treatment. Currently, few clinical trials have been conducted to evaluate the relationship between UCMSC efficacy and recipient age. Systemic inflammation is suggested to play a key role in the pathogenesis of ACLF. Studies of ACLF have shown that systemic inflammation correlates directly with the severity of the syndrome. Patients with ACLF have intense systemic inflammation and oxidative stress, unlike patients who have acute decompensation but no organ failure [30]. Thus, age-associated changes in intrahepatic immune subsets appear trivial in ACLF patients, and we did not find that recipient age affects the therapeutic effects of UCMSCs in the ACLF group. In vivo, the acute inflammatory response effectively promotes the recruitment of progenitor cells, and chronic inflammation significantly inhibits the recruitment and survival of local progenitor cells and implanted MSCs [31]. Hence, liver cirrhosis patients in this study did not have systemic inflammation, such as that observed in ACLF, and age-associated intrahepatic immune changes were hypothesized to play a key role. Chronic inflammation associated with patient age was obvious in the older liver cirrhosis group and inhibited the recruitment and survival of UCMSCs. Ultimately, UCMSC treatment for younger liver cirrhosis patients provides better efficacy than that for older liver cirrhosis patients. In the future, large-scale and prospective studies are required to optimize UCMSC treatment strategies based on age for liver cirrhosis patients.

Finally, we investigated whether UCMSCs are more suitable for acute-on-chronic liver failure or liver cirrhosis patients within the same age range. For the younger patients, only the cumulative TBIL level decrease in patients with ACLF was larger than that of patients with liver cirrhosis at week 24 after UCMSC treatment; no statistically significant differences were found between the two groups at any of the other observation weeks. Interestingly, among the older patients, ACLF patients had distinct advantages. Specifically, ALT and AST levels decreased during and after UCMSC treatment, and the most significant bilirubin decline occurred after UCMSC treatment. The factors that influence the survival and function of liver stem/progenitor cells (LSPCs) in liver aging can be divided into three categories: niches, systemic factors, and LSPC senescence [32]. Age-related alterations and niche cell aging in the extracellular matrix in the microenvironment can impede stem cell proliferation and differentiation [33]. In addition to altering the local microenvironment, aging also alters systemic factors that can profoundly impact LSPCs [14]. No younger patients aged ≥ 45 years have an increase in the expression of MSC-related genes, but MSCs have a lower differentiation potential [12]. Therefore, under the same differentiation potential conditions, as age increases, ACLF patients have a more favorable niche microenvironment and systemic factors for the differentiation of UCMSCs. Additionally, the acute inflammation associated with ACLF effectively promotes the recruitment of UCMSCs [31]. A meta-analysis [34] showed that the number of injected cells was an important factor influencing the efficacy of autologous MSC therapy. Our previous studies [10] also showed that increasing the dosage of MSCs by prolonging the treatment course can increase the curative effect of UCMSCs in liver cirrhosis patients. Therefore, the same UCMSC treatment for liver cirrhosis patients failed to achieve the same satisfactory effect as treatment for liver failure patients; liver cirrhosis patients aged ≥ 45 years especially need to be considered for treatment in personalized precision therapy.

There are some limitations of this study. First, this study was a retrospective study. Selection biases may have existed, and the study is subject to the inherent limitations associated with retrospective analyses. Second, this was a single-center study, and the sample size for the two groups was rather small. Third, the observation period was only 24 weeks; a longer observation period may provide additional insights. In summary, this was a preliminary exploration of UCMSC effects moderated by recipient age in patients with HBV-related end-stage liver disease. In the future, large-scale and prospective studies are required to confirm the exact relationship between the therapeutic effect of UCMSCs and recipient age.

In conclusion, the therapeutic effects of UCMSCs for HBV-related acute-on-chronic liver failure and liver cirrhosis varied partly by patient age. Assessing patient age is necessary prior to UCMSC clinical use. Personalized precision UCMSC therapy is crucial for patients with HBV-related end-stage liver disease.

## Supplementary Information

**Additional file 1:Supplementary Table 1.** Comparative analysis of the median decrease of UCMSCs in group A vs group B at different time points. **Supplementary Table 2.** Comparative analysis of the cumulative decreases of UCMSCs in group A vs group B at different time points. **Supplementary Table 3.** Comparative analysis of the median decrease of UCMSCs in group A vs group C at different time points. **Supplementary Table 4.** Comparative analysis of the cumulative decrease of UCMSCs in group A vs group C at different time points.

## Data Availability

The data supporting the findings of this study are available from the corresponding author upon reasonable request.

## Abbreviations

HBV: Hepatitis B virus
ALT: Alanine aminotransferase
UCMSCs: Umbilical cord mesenchymal stem cells
ACLF: Acute-on-chronic liver failure
TBIL: Total bilirubin
PTA: Prothrombin activity
MELD: Model for end-stage liver disease
AFP: Alpha fetoprotein
