# Supplementary material for: The impact of recipient age on the effects of umbilical cord mesenchymal stem cells on HBV-related acute-on-chronic liver failure and liver cirrhosis
Source: Stem Cell Res Ther. 2021 Aug 20;12:466. doi: 10.1186/s13287-021-02544-x (PMC8379867; doi:10.1186/s13287-021-02544-x)
Supplement: Supplementary file 1 — Additional file 1:Supplementary Table 1. Comparative analysis of the median decrease of UCMSCs in group A vs group B at different time points. Supplementary Table 2. Comparative analysis of the cumulative decreases of UCMSCs in group A vs group B at different time points. Supplementary Table 3. Comparative analysis of the median decrease of UCMSCs in group A vs group C at different time points. Supplementary Table 4. Comparative analysis of the cumulative decrease of UCMSCs in group A vs group C at different time points. [file 13287_2021_2544_MOESM1_ESM.docx]

**Supplementary** **Table 1.** Comparative analysis of the median decrease of UCMSCs in group A vs group B at different time points.

| **Outcome** | **Group** | **W0-W1** | | **W1-W4** | | **W4-W12** | | **W12-W24** | |
| --- | --- | --- | --- | --- | --- | --- | --- | --- | --- |
|  |  | **Value** | **P value** | **Value** | **P value** | **Value** | **P value** | **Value** | **P value** |
| **AST** | **Group A** | 18(-15 to 42.5) | NS | 7.5(-14.5 to 30.5) | NS | 10(-2 to 50) | NS | 9(-1.75 to 19.75) | NS |
|  | **Group B** | 22(4 to 104.25) |  | 4(-14 to 25) |  | 17.5(-10.2 to 42) |  | 2(-5 to 17.5) |  |
| **ALT** | **Group A** | 31(6 to 173) | NS | 15(-4.7 to 45.8) | NS | 2(-7 to 18) | NS | -4.5(-12.75 to 4.5) | NS |
|  | **Group B** | 32.5(9.7 to 335) |  | 15(-2.5 to 27.5) |  | 5.5(-9 to 18.5) |  | 0(-4 to 5.5) |  |
| **TBIL** | **Group A** | 33.5(-49 to 98.2) | NS | 72(26 to 155) | NS | 178(64 to 288) | NS | 40(19.3 to 107.5) | NS |
|  | **Group B** | 27(-6.25 to 110.7) |  | 110(27.5 to 187.5) |  | 98.5(24 to 217.5) |  | 19(-8.5 to 98) |  |
| **PTA** | **Group A** | -1(-5 to -4) | NS | 2(-2.25 to 10.3) | NS | 8.5(1.25 to 23) | NS | 0.5(-1.5 to 8.75) | NS |
|  | **Group B** | -1.5(-5.25 to 2) |  | 1(0 to 6) |  | 7(0 to 14) |  | 3.5(-1 to 13) |  |
| **MELD** | **Group A** | 0(-2 to 2) | NS | 1(-1 to 4) | NS | 6(2.5 to 10.3) | NS | 2(1 to 3.75) | NS |
|  | **Group B** | 0(-1 to 2) |  | 3(0 to 4) |  | 5.5(1 to 8.7) |  | -1(-1 to 6) |  |
| **AFP** | **Group A** | 50(8 to 122.5) | NS | -1(-12.5 to 28) | NS | -6(-39.5 to 14.5) | NS | 6(-5.5 to 44) | NS |
|  | **Group B** | 9 (1 to 85) |  | -10 (-76 to 81) |  | 3(-8 to 4) |  | 0(-2 to 0) |  |

ALT alanine aminotransferase, AST glutamic-oxaloacetic transaminase, TBIL total bilirubin, PTA prothrombin time activity, MELD model for end-stage liver disease, AFP alpha fetoprotein

**Supplementary** **Table 2.** Comparative analysis of the cumulative decreases of UCMSCs in group A vs group B at different time points.

| **Outcome** | **Group** | **W1-W0** | | **W4-W0** | | **W12-W0** | | **W24-W0** | | |
| --- | --- | --- | --- | --- | --- | --- | --- | --- | --- | --- |
|  |  | **Value** | **P value** | **Value** | **P value** | **Value** | **P value** | **Value** | **P value** | |
| **AST** | **Group A** | 18(-15 to 42.5) | NS | 17.5(-22.5 to 78.5) | NS | 35(-4 to 99.5) | NS | 67(18.7 to 103.7) | | NS |
|  | **Group B** | 22(4 to 104.25) |  | 47(-5 to 104.5) |  | 49.5(-1 to 117.2) |  | 51(8 to 213) | |  |
| **ALT** | **Group A** | 31(6 to 173) | NS | 64(-5.5 to 185.7) | NS | 71(4 to 199.2) | NS | 66.5(5.5 to 195) | | NS |
|  | **Group B** | 32.5(9.7 to 335.5) |  | 45(11 to 360.5) |  | 46(11.5 to 358.7) |  | 49(5 to 102) | |  |
| **TBIL** | **Group A** | 33.5(-49 to 98.2) | NS | 102(9.75 to 223.5) | NS | 299(159.2 to 445) | NS | 394(296 to 505.2) | | NS |
|  | **Group B** | 27(-6.2 to 110.8) |  | 166(82 to 247.5) |  | 266.5(-174 to 388.5) |  | 322(249 to 459) | |  |
| **PTA** | **Group A** | -1(-5 to -4) | NS | 4(-4 to 9) | NS | 8(0.5 to 33) | NS | 2(-1.5 to 23.2) | | NS |
|  | **Group B** | -1.5(-5.2 to 2) |  | 2(-6 to 7) |  | 5(-3 to 15) |  | 1.5(-3 to 15.7) | |  |
| **MELD** | **Group A** | 0(-2 to 2) | NS | 1(-2 to 5) | NS | 7(2 to 12) | NS | 8(3.5 to 14) | | NS |
|  | **Group B** | 0(-1 to 2) |  | 4(-1.5 to 6) |  | 4(0.2 to 9.2) |  | 3(-1 to 13) | |  |
| **AFP** | **Group A** | 50(8 to 122.5) | NS | 41(10.5 to 127) | NS | 101.5(-0.5 to 198) | NS | 114(29 to 173) | | NS |
|  | **Group B** | 9(1 to 85) |  | 87(-8 to 185) |  | 9(-2.5 to 165) |  | 20(0 to 188) | |  |

ALT alanine aminotransferase, AST glutamic-oxaloacetic transaminase, TBIL total bilirubin, PTA prothrombin time activity, MELD model for end-stage liver disease, AFP alpha fetoprotein

**Supplementary** **Table 3.** Comparative analysis of the median decrease of UCMSCs in group A vs group C at different time points.

| **Outcome** | **Group** | **W0-W1** | | | **W1-W4** | | | **W4-W12** | | | **W12-W24** | | |
| --- | --- | --- | --- | --- | --- | --- | --- | --- | --- | --- | --- | --- | --- |
|  |  | **Value** | **P value** | | **Value** | **P value** | | **Value** | **P value** | | **Value** | **P value** | |
| **AST** | **Group A** | 18(-15 to 42.5) | | NS | 7.5(-14.5 to 30.5) | | NS | 10(-2 to 50) | | NS | 9(-1.7 to 19.8) | | NS |
|  | **Group C** | 13(-10.5 to 40) | |  | 8(-12.25 to 24.5) | |  | 10(2.5 to 26) | |  | 9.5(-0.3 to 17.5) | |  |
| **ALT** | **Group A** | 31(6 to 173) | | NS | 15(-4.75 to 45.7) | | NS | 2(-7 to 18) | | NS | -4.5(-12.8 to 4.5) | | NS |
|  | **Group C** | 20(-3.5 to 153.5) | |  | 14.5(2 to 21.5) | |  | 1(-7 to 6.5) | |  | -0.5(-6.5 to 6.7) | |  |
| **TBIL** | **Group A** | 33.5(-49 to 98.2) | | NS | 72(26 to 155) | | NS | 178(64 to 288) | | NS | 40(19.2 to 107.5) | | NS |
|  | **Group C** | 46(-26 to 126.5) | |  | 75(27.2 to 155.5) | |  | 110(17.5 to 250.5) | |  | 20(6.2 to 52) | |  |
| **PTA** | **Group A** | -1(-5 to 4) | | NS | 2(-2.2 to 10.2) | | NS | 8.5(1.25 to 23) | | NS | 0.5(-1.5 to 8.8) | | NS |
|  | **Group C** | 0.5(-6.5 to 6.7) | |  | 2(-1 to 8.5) | |  | 7(3 to 17) | |  | 6(5.5 to 19) | |  |
| **MELD** | **Group A** | 0(-2 to 2) | | NS | 1(-1 to 4) | | NS | 6(2.5 to 10.2) | | NS | 2(1 to 3.75) | | NS |
|  | **Group C** | 0.5(-2 to 4) | |  | 1(-0.5 to 3) | |  | 6(1 to 10) | |  | 3(2.5 to 7.5) | |  |
| **AFP** | **Group A** | 50(8 to 122.5) | | NS | -1(-12.5 to 28) | | NS | -6(-39.5 to 14.5) | | NS | 6(-5.5 to 44) | | NS |
|  | **Group C** | 32(3 to 255) | |  | -2(-6 to 30) | |  | -2(-36.7 to 12.5) | |  | 40.5(15 to 66) | |  |

ALT alanine aminotransferase, AST glutamic-oxaloacetic transaminase, TBIL total bilirubin, PTA prothrombin time activity, MELD model for end-stage liver disease, AFP alpha fetoprotein

**Supplementary** **Table 4.** Comparative analysis of the cumulative decrease of UCMSCs in group A vs group C at different time points.

| **Outcome** | **Group** | **W1-W0** | | **W4-W0** | | **W12-W0** | | **W24-W0** | |
| --- | --- | --- | --- | --- | --- | --- | --- | --- | --- |
|  |  | **Value** | **P value** | **Value** | **P value** | **Value** | **P value** | **Value** | **P value** |
| **AST** | **Group A** | 18(-15 to 42.5) | NS | 17.5(-22.5 to 78.5) | NS | 35(-4-99.5) | NS | 67(18.7 to 103.7) | NS |
|  | **Group C** | 13(-10.5 to 40) |  | 16(-20 to 57) |  | 27(-1-77) |  | 33.5(9.5 to 85.2) |  |
| **ALT** | **Group A** | 31(6 to 173) | NS | 64(-5.5 to 185.7) | NS | 71(4-199.25) | NS | 66.5(5.5 to 195) | NS |
|  | **Group C** | 20(-3.5 to 153.5) |  | 50(7 to 172) |  | 35(0-178) |  | 54.5(-8 to 193) |  |
| **TBIL** | **Group A** | 33.5(-49 to 98.2) | NS | 102(9.75 to 223.5) | NS | 299(159.2 to 445) | NS | 394(296 to 505.3) | NS |
|  | **Group C** | 46(-26 to 126.5) |  | 120(24 to 236) |  | 253(137 to 438) |  | 288(126.5 to 462.2) |  |
| **PTA** | **Group A** | -1(-5 to 4) | NS | 4(-4 to 9) | NS | 8(0.5 to 33) | NS | 2(-1.5-23.25) | NS |
|  | **Group C** | 0.5(-6.5 to 6.7) |  | 0.5(-8 to 9) |  | 10.5(-0.5 to 25.7) |  | 0.5(-28 to 29.7) |  |
| **MELD** | **Group A** | 0(-2 to 2) | NS | 1(-2 to 5) | NS | 7(2 to 12) | NS | 8(3.5 to 14) | NS |
|  | **Group C** | 0.5(-2 to 4) |  | 0.5(-2 to 4.2) |  | 6.5(-2 to 10.5) |  | 11(8 to 12) |  |
| **AFP** | **Group A** | 50(8 to 122.5) | NS | 41(10.5 to 127) | NS | 101.5(-0.5 to 198) | NS | 114(29 to 173) | NS |
|  | **Group C** | 32(3 to 255) |  | 6(-2 to 41) |  | 1(-9 to 59) |  | 59(1.5 to 339) |  |

ALT alanine aminotransferase, AST glutamic-oxaloacetic transaminase, TBIL total bilirubin, PTA prothrombin time activity, MELD model for end-stage liver disease, AFP alpha fetoprotein
